# Genetic regulators of leaf size in *Brassica* crops

**DOI:** 10.1038/s41438-021-00526-x

**Published:** 2021-05-01

**Authors:** Umer Karamat, Xiaoxue Sun, Na Li, Jianjun Zhao

**Affiliations:** State Key Laboratory of North China Crop Improvement and Regulation, Key Laboratory of Vegetable Germplasm Innovation and Utilization of Hebei, Collaborative Innovation Center of Vegetable Industry in Hebei, College of Horticulture, Hebei Agricultural University, 071000 Baoding, China

**Keywords:** Plant molecular biology, Leaf development

## Abstract

Leaf size influences plant development and biomass and is also an important agricultural trait in *Brassica* crops, in which leaves are the main organ produced for consumption. Leaf size is determined by the coordinated regulation of cell proliferation and cell expansion during leaf development, and these processes are strictly controlled by various integrated signals from the intrinsic regulatory network and the growth environment. Understanding the molecular mechanism of leaf size control is a prerequisite for molecular breeding for crop improvement purposes. Although research on leaf size control is just beginning in *Brassica*, recent studies have identified several genes and QTLs that are important in leaf size regulation. These genes have been proposed to influence leaf growth through different pathways and mechanisms, including phytohormone biosynthesis and signaling, transcription regulation, small RNAs, and others. In this review, we summarize the current findings regarding the genetic regulators of leaf size in *Brassica* and discuss future prospects for this research.

## Introduction

*Brassica* is one of the most economically important genera of Brassicaceae. It includes a variety of horticultural crops, such as cabbage, rape, mustard, and cauliflower. *Brassica* crops are a vital source of vegetables, cooking oil, and condiments for human consumption^1,2^. Moreover, *Brassica* plants are rich in genetic diversity and phenotypic variation, providing abundant genetic resources for crop breeding and ideal materials for plant development studies^3^.

As the most important organ for photosynthesis, leaves produce the main source of energy not only for plant growth but also for human nutrition and other purposes^4,5^. The size and shape of leaves affect light energy utilization, thereby influencing plant development and biomass. In addition, leaves are critical for many physiological processes, such as photorespiration, transpiration, and temperature regulation; therefore, leaf size can also impact plant fitness and stress responses^6^. In *Brassica* vegetables, whose leaves are the main organ of consumption, the leaves are a nutrient source, providing vitamins, soluble fiber, manganese, and glucosinolates^7^. The size of the leaves of these crops is a major factor affecting plant size and yield. On the other hand, the ideal leaf size and plant size are of great importance to the appearance of these vegetables. Thus, leaf size is a key agricultural trait for these crops.

Although leaf development and growth are affected by various environmental signals, leaf size is intrinsically controlled by complex genetic networks that regulate cell proliferation and cell expansion during leaf development. After leaf primordia are initiated at the edge of the shoot apical meristem (SAM), leaves grow and reach their mature size through cell proliferation and cell expansion. In the early stage, most cells in the primordia divide continuously, resulting in a rapid increase in the cell number; in contrast, the cell size remains relatively constant^8^. Subsequently, a transition occurs in which most cell division ceases, and cell expansion predominates, thereby leading to cell enlargement^8^. Thus, the size of mature leaves is determined by both the cell number and the cell size^9–11^.

The genetic mechanisms underlying leaf size control have been extensively studied in the model plant *Arabidopsis thaliana* (*A. thaliana*), which is also a member of the Brassicaceae family. Through forward and reverse genetic studies, numerous genes involved in leaf size regulation have been identified^8,12^. These genes function in different signaling pathways or regulatory modules to influence cell proliferation and cell expansion. As there is a wide range of genomic collinearity between *A. thaliana* and *Brassica* plants^3,13^, studies in *A. thaliana* are especially helpful for understanding the mechanisms of leaf size control in *Brassica* plants and for identifying *Brassica* leaf size regulators by homolog identification. With the rapid progression of molecular studies in *Brassica* plants and the availability of genomic sequences for several *Brassica* crops, studies on leaf size control in *Brassica* crops are increasing; several genes and quantitative trait loci (QTLs) controlling leaf growth have been identified and characterized in Chinese cabbage (*B. rapa* L. ssp. *pekinensis*), cauliflower (*B. oleracea* L. var. *botrytis*), turnip (*B. rapa* L. ssp. *rapa*), and rapeseed (*B. napus*). In this review, we will discuss the leaf size regulators identified so far and highlight their possible roles in leaf growth.

## Leaf size regulators involved in phytohormone homeostasis or signaling

Phytohormones play crucial roles in plant development and responses to biotic and abiotic stresses. Recent studies have demonstrated the functions of auxin, ethylene, abscisic acid (ABA), and gibberellic acid (GA) in *Brassica* leaf growth. Several genes involved in the homeostasis of or signaling by these phytohormones have been identified as leaf size regulators in both *A. thaliana* and *Brassica* crops.

### Regulators involved in auxin signaling

Auxin biosynthesis, transport, and signaling are essential for plant growth. Auxin participates in plant development and growth by regulating cell division, cell growth, and cell differentiation. Several auxin-related genes have been found to play vital roles in maintaining the final leaf size in *Brassica* crops.

#### BrANT

*AINTEGUMENTA* (*ANT*) encodes an APETALA 2/ETHYLENE RESPONSE FACTOR (AP2/ERF) family transcription factor that responds to auxin and controls downstream gene expression to regulate organogenesis and cell proliferation^14,15^. In *A. thaliana*, *ANT* overexpression resulted in large leaves due to increased cell proliferation, indicating that *ANT* promotes leaf growth by regulating cell division^16^. In Chinese cabbage, three *ANT* and six *ANT-LIKE* (*BrAIL*) genes have been identified, and the expression of the *BrANT* genes and three of the *BrAIL* genes was responsive to auxin treatment^17^. Among these homologs, BrANT-1 shows the highest protein sequence similarity to the *A. thaliana* ANT protein (AtANT). The overexpression of the Chinese cabbage *BrANT-1* gene in *A. thaliana* increased leaf size due to enhanced cell proliferation, indicating that the effect of *BrANT-1* on leaf growth is similar to that of *AtANT*. Equally important, *A. thaliana* plants overexpressing *BrANT-1* also exhibited a significantly increased stomata number and a slightly increased net photosynthetic rate. The expression of *STOMAGEN* was also significantly enhanced in the transgenic plants. Thus, *BrANT-1* may also regulate stomatal density by upregulating the *STOMAGEN* gene^17^.

#### BrARGOS

*AUXIN-REGULATED GENE INVOLVED IN ORGAN SIZE* (*ARGOS*) is believed to function downstream of auxin and upstream of *ANT* to regulate cell proliferation and organ growth^18^. *ARGOS* encodes a protein of low molecular weight with an ORGAN SIZE RELATED (OSR) domain^19^. *ARGOS*-overexpressing *A. thaliana* plants had large leaves, while antisense *ARGOS* plants had smaller leaves than the wild-type plants^18^. Furthermore, *ARGOS* can regulate the expression of *ANT*, and the large-leaf phenotype of *ARGOS*-overexpressing plants depends on the functional *ANT*, indicating that *ANT* acts downstream of *ARGOS* to regulate organ growth^18^. The overexpression of the Chinese cabbage *BrARGOS* gene in *A. thaliana* plants increased the size of leaves and other organs due to enhanced cell proliferation^20^. Furthermore, semiquantitative RT-PCR analysis detected increased *ANT* expression in transgenic plants, revealing a conserved mechanism of *BrARGOS* for leaf size control similar to that of *AtARGOS*. Interestingly, a study on a polyploidy Chinese cabbage series revealed that the expression level of *ARGOS* was concomitantly upregulated with the increase in the ploidy level and the size of the leaves and petals, indicating that the expression of *ARGOS* is regulated by genome size^21^. However, the mechanistic basis of this regulation remains unclear.

#### pPLAIIIδ

The patatin-related phospholipase A (pPLA) family member PLAIIIδ influences organ size by regulating auxin distribution. The pPLA family enzymes hydrolyze membrane glycerolipids into lysoglycerolipids and free fatty acids and play vital roles in hormone biosynthesis and signaling, as well as other biological processes^22^. In *A. thaliana*, ten pPLAs are divided into three subfamilies, namely, pPLAI, pPLAII (α, β, γ, δ, ε), and pPLAIII (α, β, γ, δ)^23^. The overexpression of *pPLAIIIδ* in *A. thaliana* or *B. napus* resulted in shorter leaves, floral organs, and siliques but thicker stems than those of the wild type, indicating that pPLAIIIδ inhibits longitudinal growth but promotes transverse expansion during organ growth^24^. Furthermore, transgenic plants showed increased phosphatidic acid (PA) concentrations and free IAA levels in aboveground organs, revealing that pPLAIIIδ may regulate auxin distribution by regulating PA. Interestingly, the pavement cells of transgenic leaves produced fewer indentations and lobes than those of the wild type, resulting in a relatively simple network of epidermal cells and indicating that pPLAIIIδ is also involved in polar cell growth. However, the role of *Brassica* pPLAs in leaf growth has not yet been reported.

#### BrARP1 and BrDRM1

*AUXIN-REPRESSED PROTEIN 1* (*ARP1*) and *DORMANCY-ASSOCIATED PROTEIN 1* (*DRM1*) encode auxin-repressed small-peptide proteins that are conserved among several plant species. These genes have been reported to be highly expressed in nongrowing tissues and dormant buds, where they are associated with dormancy^25^. For example, *BrARP1* and *BrDRM1* from Chinese cabbage have been identified as negative regulators of plant growth. The overexpression of *BrARP1* or *BrDRM1* in *A. thaliana* plants reduced root, hypocotyl, and leaf growth due to suppressed cell elongation or cell expansion^26^. As these genes are upregulated in plants exposed to abiotic stress, it is likely that they inhibit growth, allowing plants to overcome stress by redirecting their resources.

### Regulators functioning in ethylene signaling

Ethylene is an endogenously synthesized hormone that exists in gas form. It is involved in many developmental processes and stress responses, such as dormancy, seed germination, leaf expansion, disease and pest attack, and tissue damage. It is also essential in agriculture, where it functions in organ senescence, abscission, and fruit ripening^27^. In *Brassica* crops, two important components of the ethylene signaling pathway have been recognized as leaf size regulators.

#### BrERF4

*ETHYLENE RESPONSE FACTORS* (*ERFs*) encode AP2/ERF superfamily transcription factors that are central components of the ethylene signaling pathway by regulating the transcription of ethylene-responsive genes^28^. In *A. thaliana* plants, *AtERF4* has been reported to affect many developmental and stress response processes, such as leaf senescence, anthocyanin synthesis, the plant defense response, and the iron deficiency response^29–32^. During iron deficiency, an *erf4* mutant exhibited enhanced chlorophyll content and plant growth, while the transient overexpression of *AtERF4* in *Nicotiana tabacum* leaves promoted chlorophyll degradation^32^. Similarly, overexpressing the Chinese cabbage *BrERF4* in *A. thaliana* plants reduced leaf size by inhibiting cell expansion^33,34^. The expression of two *EXPANSIN* (*EXP*) genes, *AtEX1A5* and *AtEX1A10*, was downregulated in *BrERF4*-overexpressing *A. thaliana* plants, revealing that *BrERF4* restricts cell expansion by repressing *EXP* genes^34^. However, it seems that *BrERF4* regulates the expression of these genes indirectly, as no ERF binding site was identified in the promoter regions of *AtEX1A5* and *AtEX1A10*. In addition, it has been demonstrated that the *ERF4* gene from Chinese cabbage and other plant species is also involved in stress tolerance and disease resistance^33,35,36^. It will be of great importance to study how ERF4 mediates stress-induced growth retardation by reducing cell expansion.

#### BoCDAG1

*CURD DEVELOPMENT ASSOCIATED GENE 1* (*CDAG1*) encodes a protein with an OSR domain. It was identified as a growth stimulator in cauliflower due to its high expression in young cauliflower curds^19^. The overexpression of *CDAG1* in *A. thaliana* plants increased the size of leaves, flowers, roots, and other organs. Similarly, the ectopic expression of *CDAG1* in cauliflower promoted plant growth and increased plant biomass. Therefore, *CDAG1* could be an ideal target for the genetic engineering of high-yield crops. Although CDAG1 is homologous to *A. thaliana* ARGOS and ARGOSE-LIKE (ARL), which also contain an OSR domain, these three proteins seem to play different roles in organ growth regulation. Specifically, ARGOS promotes organ growth by enhancing cell proliferation^18^, while ARL mainly influences cell expansion;^37^ by contrast, *CDAG1*-overexpressing plants showed increased cell number and cell size^19^. A promoting effect on both cell proliferation and cell expansion was also reported for another OSR family gene, namely, *OSR1*^38^, in *A. thaliana*. Unexpectedly, the overexpression of *CDAG1* upregulates several *ERF* genes, whereas *BrERF4* overexpression inhibits cell expansion and leaf growth^33,34^. Further studies are needed to clarify the mechanism of *CDAG1* in growth regulation.

### Regulators involved in abscisic acid (ABA) and gibberellic acid (GA) signaling

#### BnNCED3

ABA is a phytohormone associated with various stress responses. It regulates many developmental and physiological processes, such as osmosis, seed development, seed germination, leaf senescence, stomatal closure, and bud dormancy. Although defects in vegetable growth have been reported in several ABA-deficient mutants, ABA is generally considered to be a growth inhibitor under stress conditions. For example, it restricts leaf growth by repressing vegetative leaf emergence, promoting flowering, and accelerating leaf senescence^39^. Activating *AtNCED3*, which encodes a key enzyme of ABA biosynthesis, enhanced ABA biosynthesis, restricted plant growth, and enhanced drought tolerance^40–42^. The ortholog of AtNCED3 from *B. napus*, BnNCED3, has been demonstrated to play a similar role in repressing plant growth. The overexpression of *BnNCED3* in *A. thaliana* plants increased ABA biosynthesis and enhanced abiotic stress tolerance and leaf senescence, indicating the importance of ABA in integrating environmental cues and developmental signals to regulate leaf senescence^43^.

#### BrRGA1

GA is a plant hormone that functions in various growth and developmental processes, including leaf expansion, stem elongation, flowering transition, and seed germination^44^. GA-deficient and GA-insensitive mutants generally show a dwarf phenotype and have been extensively used in plant breeding to improve lodging resistance. The famous “Green Revolution” gene in wheat encodes a central repressor of GA signaling named DELLA^45^. DELLAs are plant-specific GRAS family transcription factors that repress GA-responsive genes. GA perception leads to the degradation of DELLA proteins and, therefore, the activation of downstream genes^44,46^. The widely utilized modern semidwarf wheat cultivars carry the *Reduced height-1* allele that encodes a mutant form of the DELLA protein, which causes GA insensitivity and decreased stem elongation^47^. DELLA proteins are highly conserved among dicotyledon and monocotyledon species, and different paralogs play distinct but somewhat overlapping roles in the GA response^44,46^. The *A. thaliana* genome encodes five DELLA proteins. Mutations leading to DELLA stabilization resulted in dwarf phenotypes, whereas the loss of function of four of the DELLA proteins promoted leaf growth through both cell division and cell expansion^48,49^. In *B. rapa*, a semidominant dwarf mutant, *dwf2*, was insensitive to exogenous GA3^50^. Comparative mapping analysis showed that *DWF2* encodes BrRGA1 (Bra024875), which is homologous to the *A. thaliana* DELLA protein RGA (repressor of *ga1-3*). The mutant Brrga1-d protein has an amino acid substitution in a conserved motif required for protein degradation, thereby leading to the accumulation of the protein^51^. Although DELLA proteins were shown to restrain leaf growth in *A. thaliana*, the *Brrga1-d* mutant has not been reported to affect leaf size. It is unclear whether other DELLA proteins in *B. rapa* function redundantly with *BrRGA1* to regulate leaf growth.

### Control of leaf growth in *Brassica* through the integration of hormone signaling

**Fig. 1:**
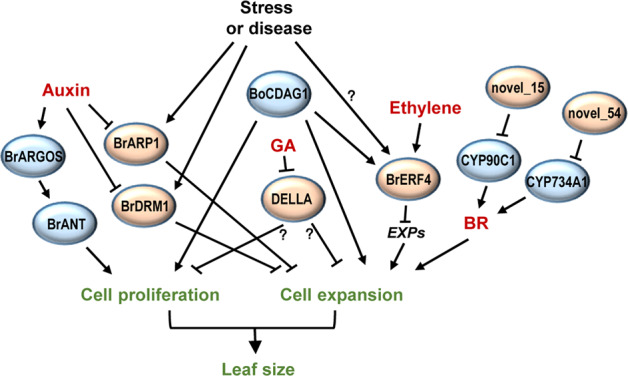
Control of leaf size in *Brassica* through the integration of hormone signaling. Leaf size is influenced by auxin, ethylene and brassinosteroids (BRs). The role of GA in leaf size control in *Brassica* remains unclear. The positive and negative regulators are shown in blue and yellow ovals, respectively. Arrows indicate positive regulation, and T indicates negative regulation. Question marks indicate undefined regulation

Based on the current findings, we propose a possible model of leaf size control through integrated hormone signaling (Fig. 1). First, auxin promotes leaf growth by inducing *BrARGOS* and *BrANT* to accelerate cell proliferation and repressing *BrARP1* and *BrDRM1* to enhance cell expansion^17,20,26^. Meanwhile, ethylene restricts cell expansion and leaf growth through BrERF4 by downregulating *EXP* genes^33,34^. Although the expression of some *ERF* genes is induced by BoCDAG1, BrERF4 and BoCDAG1 have the opposite effect on leaf size^19,33,34^. Therefore, it remains unclear whether BoCDAG1 functions through ethylene signaling to control leaf growth. In addition, brassinosteroids (BRs) promote cell expansion and leaf growth^52^ (as discussed in “MicroRNAs that regulate leaf size”). In *Arabidopsis*, GA positively regulates leaf growth through both cell proliferation and cell expansion. The loss of function of the GA signaling-repressing DELLA proteins leads to enlarged leaf size^48,49^. In *B. rapa*, the mutation of the DELLA protein BrRGA1 causes a dwarf phenotype;^50,51^ however, the role of *Brassica* DELLA proteins in leaf size control remains poorly understood.

Notably, several negative regulators of leaf size have been shown to respond to stress or disease. For instance, the expression of *BrARP1* and *BrDRM1* can be induced by abiotic stresses^26^, and the *ERF4* genes from Chinese cabbage and other plant species are also involved in stress tolerance and disease resistance^33,35,36^. It is likely that under stress or disease conditions, these genes can mediate signals to restrict leaf growth. Furthermore, increasing ABA biosynthesis through the overexpression of *BnNCED3* can enhance abiotic stress tolerance and leaf senescence^43^, indicating that ABA can accelerate leaf senescence under abiotic stress.

## Other transcription factors that regulate leaf size

Transcription factors contain a specific DNA binding domain and a transcriptional regulation region. They bind to the *cis*-regulatory elements of their target genes to repress or activate gene transcription. Transcription factors play key roles in many developmental and physiological processes by controlling gene expression. In addition to the aforementioned transcription factors, several other transcription factors have been found to regulate leaf growth.

### BrrTCP2

Members of the TCP family of plant-specific transcription factors contain a 59-amino acid basic helix–loop–helix (bHLH) motif defined as the TCP domain, which is named after TEOSINTE BRANCHED1 (TB1) from maize (*Zea mays*), CYCLOIDEA (CYC) from *Antirrhinum majus*, and PROLIFERATING CELL FACTORS (PCFs) from rice (*Oryza sativa*)^53^. Numerous *TCP* genes have been identified in various plant species, and TCP proteins are grouped into two classes according to the subtle differences in their TCP domains. Class I TCPs have been proposed to promote plant growth, while Class II TCPs have been reported to prevent cell proliferation and plant growth^53^. The *A. thaliana* genome encodes 24 TCP proteins. TCP2, TCP3, TCP4, TCP10, and TCP24 are class II TCPs that function redundantly in leaf growth. The loss of function of these genes resulted in enlarged leaves and wrinkled leaf margins due to excessive cell division. In turnip, 39 TCPs have been identified^54^. The overexpression of *BrrTCP2* rescued the phenotype of the *A. thaliana tcp2*/*4*/*10* triple mutant, and the overexpression of *BrrTCP2* in wild-type *A. thaliana* plants reduced the cell number and leaf size, indicating that *BrrTCP2* limits leaf growth by inhibiting cell proliferation^54^. In addition, miR319a-targeted *BrpTCP4* has been reported to regulate the size and shape of heads of Chinese cabbage (refer to the MicroRNAs section for details).

### BrGRFs

Members of the GROWTH-REGULATING FACTOR (GRF) family of small, plant-specific transcription factors function in growth and development^55,56^. GRFs form protein complexes with the transcriptional coactivators GRF-INTERACTING FACTORS (GIFs)^55,56^. In *A. thaliana*, nine *GRF* and three *GIF* genes have been identified. Although most GRFs have been found to positively regulate leaf size, they seem to play different roles in leaf growth. For example, AtGRF1–3 function redundantly to enhance cell expansion^57^, while AtGRF4 functions in cell proliferation in leaves and modulates the development of the SAM and cotyledons;^58^ by contrast, AtGRF5 interacts with GIF1/ANGUSTIFOLIA3 (AN3) to enhance cell proliferation in leaf primordia^59^. Genome-wide analysis identified 17 *GRFs* in Chinese cabbage^60^. The overexpression of *BrGRF3-1*/*3–2*/*5*/*7*/*8–1*/*8–2*/*9* in *A. thaliana* plants increased the size of cotyledons, leaves, flowers, siliques, and seeds, as well as the seed oil content. Furthermore, the enhanced organ growth of the transgenic plants resulted from increased cell proliferation but not cell expansion, indicating that these *BrGRFs* stimulate organ growth by promoting cell proliferation^61^. In *A. thaliana* plants, GRF1–4 and GRF7–9 are targeted by miR396, and the expression of miR396 is regulated by TCP4^62,63^. However, it is unclear whether this regulatory module exists in *Brassica* species.

### BrNGA1

NGATHA (NGA) proteins belong to the plant-specific B3 superfamily^64^. NGA1–4 from *A. thaliana* form a small protein subgroup and play key roles in leaf and flower development. The quadruple mutant (*nga1 nga2 nga3 nga4*) produces wide and serrated leaves and abnormal flowers, while the overexpression of these genes reduces leaf growth^65,66^. The *B. rapa* genome encodes four NGA1, three NGA2, two NGA3, and two NGA4 proteins. The overexpression of *BrNGA1* in *A. thaliana* resulted in small and narrow leaves. Kinematic analyses confirmed that *BrNGA1* affected both the rate and the duration of cell proliferation in the leaves. Consistently, low levels of *CycB1;1* and *CycD3;1* were observed in leaves overexpressing *BrNGA1*, suggesting that *BrNGA1* regulates organ growth by limiting cell proliferation^67^. In *A. thaliana* plants, NGA-like proteins (NGALs) have been shown to restrict organ growth by repressing (*KLUH*) *KLU* expression^68^. The role of KLU in organ growth has not been reported in *Brassica* species.

## MicroRNAs that regulate leaf size

MicroRNAs (miRNAs) are endogenous RNA sequences of ~22 nucleotides that are key components of gene regulatory networks in plants and animals^69^. Pre-miRNAs are transcribed from the corresponding miRNA genes and processed by several endonucleases to yield mature miRNAs that are subsequently incorporated into the RNA-induced silencing complex (RISC). miRNAs mediate the targeting of RISC to the corresponding mRNAs and promote the degradation of target mRNAs^70^. In *A. thaliana* plants, several miRNAs have been reported to play key roles in regulating leaf growth^63,71,72^. Some evidence suggests that miRNAs are also important leaf size regulators in *Brassica* crops.

### MiR319

MiR319 is a key regulator of leaf development that targets *TCP* genes^71,72^. In the *A. thaliana jaw-D* mutant, the increased expression of miR319 downregulated *TCPs*, resulting in large and highly wrinkled leaves. In Chinese cabbage, miR319a has been demonstrated to regulate the size and shape of the cabbage heads^73^. Furthermore, the overexpression of *BrpMIR319a2* in Chinese cabbage altered the expression pattern of *BrpTCP4*, leading to the excess growth of both apical and interveinal regions and resulting in enlarged and cylindrical heads.

### MiR394

In *A. thaliana* plants, miR394 and its target gene *LEAF CURLING RESPONSIVENESS* (*AtLCR*) are essential regulators of leaf morphology and stem cell maintenance^74,75^. The *lcr* mutant and *miR394*-overexpressing plants were semidwarf with large and upward curling leaves. In contrast, the overexpression of miR394-resistant *LCR* resulted in the downward curling of leaves^74^. In rapeseed, the overexpression of *miR394* downregulated *BnLCR*, delayed flowering time and increased the size of leaves, pods, and seeds^76^. In addition, transgenic rapeseed overexpressing the antisense mRNA of *BnLCR* produces large leaves, and that overexpressing *BnLCR* exhibits decreased leaf size. These studies revealed that miRNA394 and *BnLCR* play conserved functions in regulating leaf growth in rapeseed^76^. It is unclear whether *BnLCR* regulates cell proliferation or cell expansion processes in leaf growth.

### Novel_15 and novel_54

In Chinese cabbage, small leaf and plant sizes were observed in autotetraploid plants (4X) created from a doubled haploid (2X) line. Transcriptome analysis showed that two miRNAs, novel_15 and novel_54, were upregulated and that their target genes (*BraA01000252* and *BraA05004386*, encoding CYP90C1 and CYP734A1, respectively), which are both involved in BR biosynthesis, were downregulated. Consistently, the autotetraploid plants showed low BR levels compared with the haploid line, indicating that these miRNAs may be involved in leaf growth through their regulation of BR biosynthesis^52^.

### MiRNA regulatory network for leaf size control

**Fig. 2:**
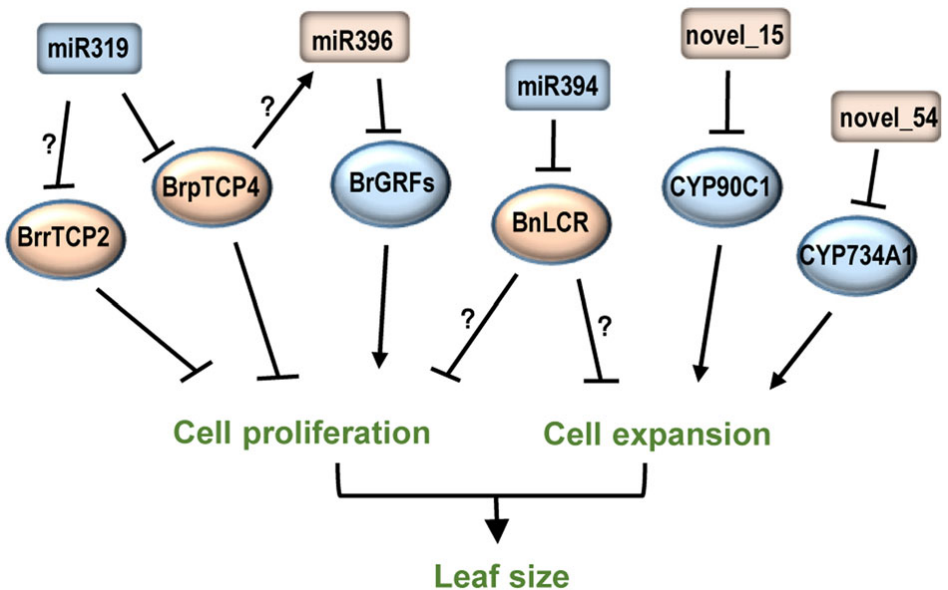
MiRNAs and their targets involved in *Brassica* leaf size regulation. MiRNAs are presented in rectangles, and their targets are presented in ovals. The positive and negative regulators are shown in blue and yellow, respectively. Arrows indicate positive regulation, and T indicates negative regulation. Question marks indicate undefined regulation

The function of miRNAs and their targets in leaf size control has been extensively studied in the model plant *A. thaliana*. The miR319-TCPs-miR396-GRFs module plays central roles in regulating leaf growth. In this module, class II TCP transcription factors restrict cell proliferation during leaf growth, while GRFs promote cell division. TCPs and GRFs are targeted by miR319 and miR396, respectively. Meanwhile, TCPs can regulate the expression of miR396 to modulate the transcription of GRFs. Some evidence suggests that this regulatory module is likely conserved in *Brassica* (Fig. 2). In Chinese cabbage, miR319a-targeted *BrpTCP4* has been found to regulate the size of cabbage heads, and *BrrTCP2* from turnip was also identified as a negative regulator of leaf size. However, it remains unknown whether *BrrTCP2* is targeted by miR319 and whether *Brassica* TCPs can regulate the expression of miR396. Another miRNA, miR394, targets LCR to regulate leaf growth in rapeseed and is similar to that in *A. thaliana*. Moreover, two novel miRNAs, novel_15 and novel_54, were identified as negative leaf size regulators targeting two genes involved in BR biosynthesis^52^. In combination, miRNAs and their targets are key regulators controlling leaf growth in *Brassica* (Fig. 2).

## Regulators involved in other pathways

### BnDA1

*DA1* encodes a ubiquitin-dependent protease that negatively regulates organ size^77,78^. The *A. thaliana da1-1* mutant produced large leaves, flowers, and seeds. The *da1-1* allele encodes a mutant protein with an arginine-to-lysine substitution at position 358 (DA1^R358K^). This mutant protein has a negative effect on DA1 and its homolog, DA1-related (DAR1), in *A. thaliana* by prolonging the duration of cell proliferation and increasing the size of organs. The overexpression of *DA1*^*R358K*^ in wild-type *A. thaliana* plants increased organ size, which mimicked the phenotype of plants with a simultaneous disruption of *DA1* and *DAR1*^77^. In contrast, the overexpression of wild-type *DA1* inhibited plant growth^79^. The BnDA1 protein from *B. napus* is highly homologous to *A. thaliana* DA1, with 83.15% amino acid sequence similarity. The overexpression of *BnDA1* restored the phenotype of the *A. thaliana da1-1* mutant, and *B. napus* plants overexpressing *AtDA1*^*R358K*^ showed increased seed, cotyledon, leaf, flower, and silique size as well as increased biomass. Furthermore, candidate gene association analyses suggested that *BnDA1* also contributes to seed weight^80^. These studies demonstrated that DA1 has a conserved function related to organ size control in *Brassica* species and can be used for biomass improvement. In *A. thaliana* plants, the protease activity of DA1 can be activated by ubiquitination, and active DA1 can cleave the TCP family transcription factors TCP14/15 to regulate organ growth^78,81^. It would be worthwhile to investigate whether BrDA1 acts through BrTCP transcription factors to regulate leaf size in *Brassica* species.

### FCA-RRM

*FLOWERING CONTROL LOCUS A* (*FCA*) is a key regulator of floral transition^82^. It regulates the expression of the floral repressor *FLOWERING LOCUS C* (*FLC*) by affecting the alternative polyadenylation of *FLC* antisense transcripts^83,84^. The FCA protein contains two RNA-binding domains (RRMs) that are highly conserved among different plant species^85^. Intriguingly, these RRM domains have been reported to promote plant growth. The overexpression of FCA-RRM1 and FCA-RRM2 can increase cell size in rice and cotton, thereby improving the yield^85–87^. Similarly, *B. napus* plants overexpressing *B. napus* FCA-RRM2 exhibited increased cell size and organ size. Notably, the expression of the *cyclin-B2-1* gene was remarkably downregulated in transgenic rapes overexpressing FCA-RRM2. However, whether the increase in cell size is caused by the decreased expression of *cyclin-B2-1* remains elusive^88^.

### BrPHYB

Phytochrome B (PHYB) is a red-light receptor that mediates light signaling and plant development. It is involved in various developmental processes, such as seed germination, organ growth, flowering, and the shade-avoidance response^89^. The loss of function of PHYB in *A. thaliana* and other plants led to constitutive symptoms of shade-avoidance syndrome (SAS), such as longer internodes, taller plants, and lower tiller numbers^90–92^. In contrast, the overexpression of *PHYB* inhibited hypocotyl elongation^93^. A recent study demonstrated that ectopic expression of the Chinese cabbage *PHYB* gene in *A. thaliana* plants decreased leaf length and plant height. Surprisingly, *AtPHYB*-overexpressing plants flowered earlier than wild-type plants, whereas the overexpression of *BrPHYB* in *A. thaliana* caused late flowering under short-day conditions. Further analyses showed that *BrPHYB* likely delayed flowering by repressing the expression of gibberellin biosynthesis genes, while *AtPHYB* promoted flowering by upregulating *FLOWERING LOCUS T*; these results indicate that *BrPHYB* and *AtPHYB* have conserved functions with regard to cell elongation but divergent roles with regard to flowering time^94^.

## QTLs of leaf size control

QTLs are genomic regions that influence the inheritance of quantitative traits. In recent years, QTL mapping has been widely used to identify candidate genes for leaf traits in different crops, such as tomato, maize, grape, and *A. thaliana*^95–98^. In *Brassica*, a number of QTLs of leaf traits have been identified using different mapping populations. Some of these QTLs colocalized with genes whose homologs in *A. thaliana* have been identified as key regulators of leaf development and growth. These studies provided useful information for further identification of leaf size regulators in *Brassica*.

Using F2 populations derived from crosses of rapid-cycling *Brassica* to three *B. oleracea* varieties, 47 QTLs influencing plant size were detected by restriction fragment length polymorphisms (RFLPs). The QTLs related to lamina length were found to correspond to five ancestral genes, namely, *REVOLUTA* (*REV*), *AUXIN RESISTANT 1* (*AXR1*), *AXR3*, *AXR4*, and *ASYMMETRIC LEAVES 2* (*AS2*), which are essential for leaf initiation and morphogenesis in *A. thaliana* plants^99^.

In *B. rapa*, three QTLs for plant height and ten QTLs for leaf traits were detected by analyzing three different types of populations developed from wide crosses between *B. rapa* accessions^100^. Similarly, three QTLs for leaf blade length and three QTLs for leaf blade width were identified by using different populations constructed from two Chinese cabbage inbred lines, ‘Chiifu-402-42’ and ‘Kenshin-40-43’; these QTLs coincided with *CYCB2;4, CYCD3;1, ULT1, AN3*, and *ANT*, which are involved in cell cycle and leaf growth regulation^101^. By using a doubled haploid population derived from a cross between the oil type cultivar yellow sarson (ssp. *trilocularis*) and the vegetable type cultivar pak choi (ssp. *chinensis*), 167 QTLs for leaf traits were detected. Several QTLs for leaf size colocalized with the key regulators of leaf development in *A. thaliana*, including *BrGRF5*_*A01, ASYMMETRIC LEAVES1* (*BrAS1*)_*A03, BrFLC5*_*A03*, *LONGIFOLIA1* (*BrLNG1*)*_A10, PINHEAD* (*BrPNH*)*_A09*, *SQUAMOSA PROMOTER BINDING PROTEINLIKE5* (*BrSPL5*)*_A01*, and *BrSPL5_A05*. Transcript analysis showed that the expression of *BrLNG1_A10* was positively correlated with leaf size. Genetic regulatory network analysis revealed that *BrCycB2;4_A07*, *BrARGOS_A07*, *BrARL_A03*, and *BrCYCB1_A01* were also associated with leaf size and plant architecture^102^.

In *B. napus*, 31 QTLs related to leaf morphology traits have been identified using recombinant inbred lines (RILs) constructed from the parental lines GH06 and P174. For leaf size regulation, eight QTLs for petiole length, two QTLs for lamina width, five QTLs for lamina length, eight QTLs for lamina size ratio, and two QTLs for total leaf size were detected. Gene expression analyses revealed that several regulators of leaf shape and size were differentially expressed in lines with distinct leaf shapes, including *AS2*, *gibberellin 20-oxidase 3* (*GA20OX3*), gibberellin-regulated family genes, *GRF*, and *KNOTTED1-like homeobox genes* (*KNATs*)^103^.

## Conclusion and future prospects

**Fig. 3:**
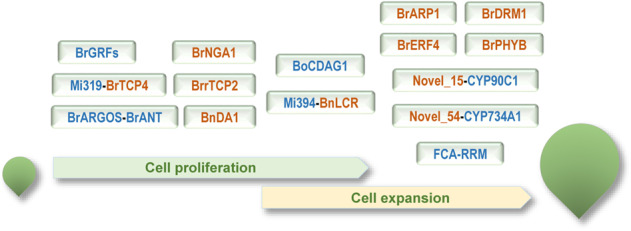
Identified leaf size regulators in *Brassica*. Positive and negative regulators are shown in blue and orange, respectively

The size of a mature leaf is regulated by two partially overlapping processes, namely, cell proliferation and cell expansion. For *Brassica* crops, especially for leafy vegetables, leaf size influences not only plant biomass and crop yield but also plant size and appearance quality. Although recent studies have identified several genetic regulators of leaf size in *Brassica* species, our knowledge of leaf size control in *Brassica* crops is still poor. In *A. thaliana* plants, nearly a hundred genes have been found to influence leaf size;^8,12^ by contrast, only a small portion of the corresponding *Brassica* homologs have been characterized (Fig. 3, Table 1), implying that a large number of leaf size regulators have yet to be discovered. On the other hand, a large proportion of the *Brassica* leaf size regulators reported so far have been identified through homologous gene identification. Due to the unavailability of transgenic technology, *A. thaliana* transgenic lines were used to verify gene function in most cases. Although there is evidence that the leaf size control functions of these genes are largely conserved, the effects of the loss of function or overexpression of these genes in *Brassica* plants are uncertain. Compared with *A. thaliana*, *Brassica* plants have experienced a whole-genome triplication (WGT) event, so that numerous *A. thaliana* genes have multiple copies in *Brassica* species. Furthermore, it was reported that phytohormone-responsive genes were significantly overretained in the *B. rapa* and *B. oleracea* genomes^3^. The complexity of *Brassica* genomes may have contributed to the abundant morphological diversity of *Brassica* plants and may also have created more complicated and species-specific genetic networks for leaf size regulation. Therefore, there is an urgent need to investigate the role of the identified regulators of leaf development in different *Brassica* crops.

**Table 1:** Leaf-size regulators identified in *Brassica* crops

Species	Gene	Gene/protein family	Positive/negative regulator	Gene ID	Gene ID in *Arabidopsis*	Reference
*B. rapa* L. ssp. *Pekinensis*	*BrANT-1*	AP2/ERF family	Positive	Bra017852	AT4G37750	^17^
*B. rapa* L. ssp. *Pekinensis*	*BrARGOS*	OSR-domain containing	Positive	Bra003394	AT3G59900	^20^
*B. rapa* L. ssp. *Pekinensis*	*BrDRM1*	Dormancy-associated protein	Negative	Bra032894	AT1G28330	^26^
*B. rapa* L. ssp. *pekinensis*	*BrARP1*	Auxin-repressed protein	Negative	Bra022955	At2G33830	^26^
*B. rapa* L. ssp. *pekinensis*	*BrERF4*	AP2/ERF family	Negative	Bra001588	AT3G15210	^33,34^
*B. oleracea* L. var. *botrytis*	*BoCDAG1*	OSR-domain containing	Positive	Bol021661	AT2G44080	^19^
*B. napus*	*BnNCED3*	9-cis-epoxycarotenoid dioxygenase	Promotes leaf senescence	BnaA03g33400D	AT3G14440	^43^
*B. rapa* L. ssp. *Rapa*	*BrrTCP2*	TCP family	Negative	Bra012600	AT4G18390	^54^
*B. rapa* L. ssp. *pekinensis*	*BrGRFs*	GRF family	Positive	Bra023066, Bra005268	AT2G36400	^61^
*B. rapa*	*BrNGA1*	B3 family	Negative	Bra097255	AT2G46870	^67^
*B. rapa* L. ssp. *pekinensis*	*miR319a*	MicroRNA	Positive	GenBank ID KJ130320	AT4G23713	^73^
*B. napus*	*miR394*	MicroRNA	Positive	BnaC05g21250D	AT1G27340	^76^
*B. napus*	*LCR*	F-box protein	Negative	GenBank ID CDX84930.1	AT1G27340	^76^
*B. napus*	*DA1*	Ubiquitin-dependent protease	Negative	BnaC05g14930D	AT1G19270	^80^
*B. napus*	*FCA-RRM2*	RRM domain	Positive	BnaC01g21860D	AT5G54580	^88^
*B. rapa* L. ssp. *pekinensis*	*BrPHYB*	Red light receptor	Negative	Bra022192	AT2G18790	^94^

The development of whole-genome sequencing technology and the MutMap method has enabled the identification of novel regulators by genome-wide association studies or mutant analyses. Currently, these studies are beginning to emerge in *Brassica* crops and are expected to make rapid progress at the forefront of functional genomics. In addition, the establishment of efficient genetic transformation methods and the use of gene-editing technology will help to further verify the function of these regulators. The next challenge is to clarify the functionally redundant and divergent roles of the multiple copies of regulators and the interactions between different regulators and finally to integrate the regulatory networks in different *Brassica* species. With the development of molecular breeding technologies, it has become possible to modulate the agricultural traits of crops by selecting or manipulating target genes. Identifying leaf size regulators in *Brassica* crops and understanding their function will inform the improvement of *Brassica* crops through the breeding high-yield crops with optimal leaf sizes as well as novel vegetable varieties with ideal leaf and plant sizes.
